# Facilitating Energy and Charge Transfer from CsPbBr_3_ Perovskite Nanocrystals via Ligand Shell Reconstruction

**DOI:** 10.1021/acsami.5c03095

**Published:** 2025-05-15

**Authors:** Aaron Malinoski, Jingheng Yuan, Chen Wang

**Affiliations:** † Department of Chemistry and Biochemistry, Queens College, CUNY, Flushing, New York 11367, United States; ‡ The Graduate Center of CUNY, New York, New York 10016, United States

**Keywords:** lead-halide perovskite nanocrystals, surface functionalization, energy transfer, photoinduced charge transfer, transient absorption

## Abstract

Efficiently extracting photon energy from colloidal lead halide perovskite nanocrystals (PNCs) as excitons and charge carriers is a crucial step in many applications of these materials. We herein report a functionalization strategy based on reconstructing the surface chemical environment of CsPbBr_3_ PNCs to strengthen the binding of acceptor motifs and, thereby, enhance energy and charge carrier transfer efficiency. A zwitterion ligand, 2-ammonium benzenesulfonate, was employed to protect the integrity of the PNC surface during a purification step for removing excess original synthetic ligands. Heterocyclic-carboxylate structures with strong chelating binding effects were utilized as the anchoring motifs to couple the acceptors to the PNC surface. Compared to directly applying the acceptors to as-synthesized PNCs, the new method achieved at least a 6-fold increase in transportation efficiency for both an oligothiophene triplet energy acceptor and a quinoline-derivative electron acceptor. NMR spectroscopy systematically analyzed the binding conditions of different surface ligands in each step of functionalization. The improved functionalization was attributed to the diminishment of competitive adsorption after the purification step. We identified the N-heterocyclic-carboxylate structure as the most effective anchoring group. Transient absorption spectroscopy was employed to monitor the triplet energy transfer and charge carrier migration processes in the PNC-acceptor complexes and evaluate their rate constants. Spectral and dynamic features for distinguishing the electron transfer process from triplet energy transfer were summarized. Our surface reconstruction strategy will benefit the development of PNC-based optoelectronics and promote the application of perovskite materials as photosensitizers in different photophysical and photochemical processes.

## Graphical abstract

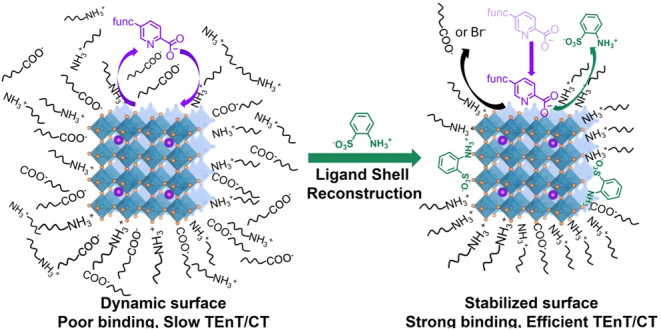

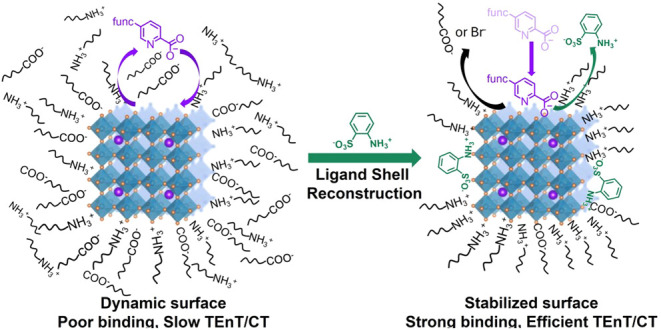

## Introduction

This paper reports a surface functionalization strategy to couple molecular acceptors on the surface of CsPbBr_3_ perovskite nanocrystals (PNCs) for facilitating energy and charge carrier transfer. All-inorganic lead halide PNCs are promising light-harvesting materials for their superior optical absorbability and tunable bandgap energy. PNCs have been employed to develop solar cells,^1^ and serve as important photosensitizers in photon energy upconversion^2–5^ and photocatalytic reactions.^6^ All these applications require facile extraction of photogenerated excitons or charge carriers from PNCs to the target acceptors that carry out the desired work. Promoting exciton and carrier migration across the PNC surface also benefits the development of PNC-based optoelectronics, such as photovoltaic devices, photon detectors, and light-emitting diodes.^7–11^

Like conventional semiconductor nanocrystals, PNCs confront the dilemma of protection versus insulation. To maintain stability and passivate defects, the PNC surface is often capped by ligands with long aliphatic chains, but the inert ligand shell poses obstacles for excitons and charge carriers to transport.^12^ It is thus pivotal to mediate PNCs’ surface chemistry to provide accessibility to acceptors and enhance their electronic coupling.^13–18^ A common strategy involves directly grafting acceptors to the PNC surface to realize close contact for maximizing transfer efficiency.^19–21^ The attached small molecular acceptors can also serve as shuttles to further transport excitons as well as charge carriers to their destination.^2,3,22,23^ Ideally, we will resolve the dilemma in various application scenarios if the surface ligand shell can be flexibly functionalized with different acceptors without affecting its integrity.

However, PNCs differ from other semiconductor nanocrystals for their highly ionic perovskite lattice, which results in a dynamic surface ligand shell,^24^ making it challenging to firmly attach functional molecules. Most previously reported surface functionalization strategies employed carboxylate or ammonium as anchoring groups. The former is known for its weak coordination to the Pb (II) sites,^25^ and the latter binds to the PNC surface through non-covalent interactions.^24^ The weak binding affinity of these anchoring groups causes the need for excess acceptors to ensure transportation efficiency. Candidates of good anchoring motifs can be found from earlier studies on surface passivation of PNCs: many good passivation ligands, including soft Lewis bases,^25–27^ chelating ligands,^28–30^ and zwitterionic binding groups,^31–33^ were identified as strongly bound structures. However, our prior research on surface functionalization found that the stability of grafted functional molecules can be seriously affected by the competitive adsorption from the original synthetic ligands in the solution.^28,32^ Unfortunately, it is often necessary to accompany PNCs with an excess amount of synthetic ligands after synthesis to maintain their dimension and protect the vulnerable surface,^34,35^ which worsens the surface accessibility. Therefore, it is essential to develop a post-synthetic treatment to purify PNCs before attaching functional molecules.

We herein present a two-step functionalization procedure to facilitate the binding of triplet energy and electron acceptors to the PNC surface and demonstrate the rise of transportation efficiency using steady-state and time-resolved optical spectroscopy. First, we implemented a purification step utilizing protective ligands to reconstruct the surface chemical environment and suppress competitive adsorption. Second, we utilized the strong chelating coordination of heterocyclic carboxylate ligands to anchor oligothiophene and quinoline derivatives as model acceptors on the PNC surface. The chelating effect of quinoline-based ligands has been utilized to couple triplet energy acceptors to the surface of InP nanocrystals to realize photon upconversion and produce delayed photoluminescence.^36^ Bidentate 8-hydroxyquinoline has been employed to mediate the electron transfer process from PNCs.^37^ Polythiophene derivatives are well-known organic semiconductors and have been incorporated in perovskite nanostructures for fabricating composite materials.^38–42^ Our time-resolved optical spectroscopic measurements characterized the energy and carrier transfer processes in the complexes of PNCs and model acceptors. The rates of these transportation processes are quantified to evaluate the effect of the surface engineering strategy.

## Results and Discussion

### A Two-Step Functionalization Strategy for Grafting Molecular Acceptors to the PNC Surface

The schematic illustration of the functionalization strategy is shown in Figure 1A. The excess synthetic ligands in the as-synthesized PNCs were removed using a purification method adapted from a previous study.^32^ Using 2-ammonium benzenesulfonate ligands (2ABS) as the protective ligands, the narrow size distribution and the strong quantum confinement of PNCs were well-preserved after purification, as shown by the UV–vis absorption and photoluminescence spectra in Figure S1. The 2ABS-treated PNC samples maintained high photoluminescence quantum yields (PLQY, Φ_PL_) between 0.61 and 0.89 for different batches.

**Figure 1:**
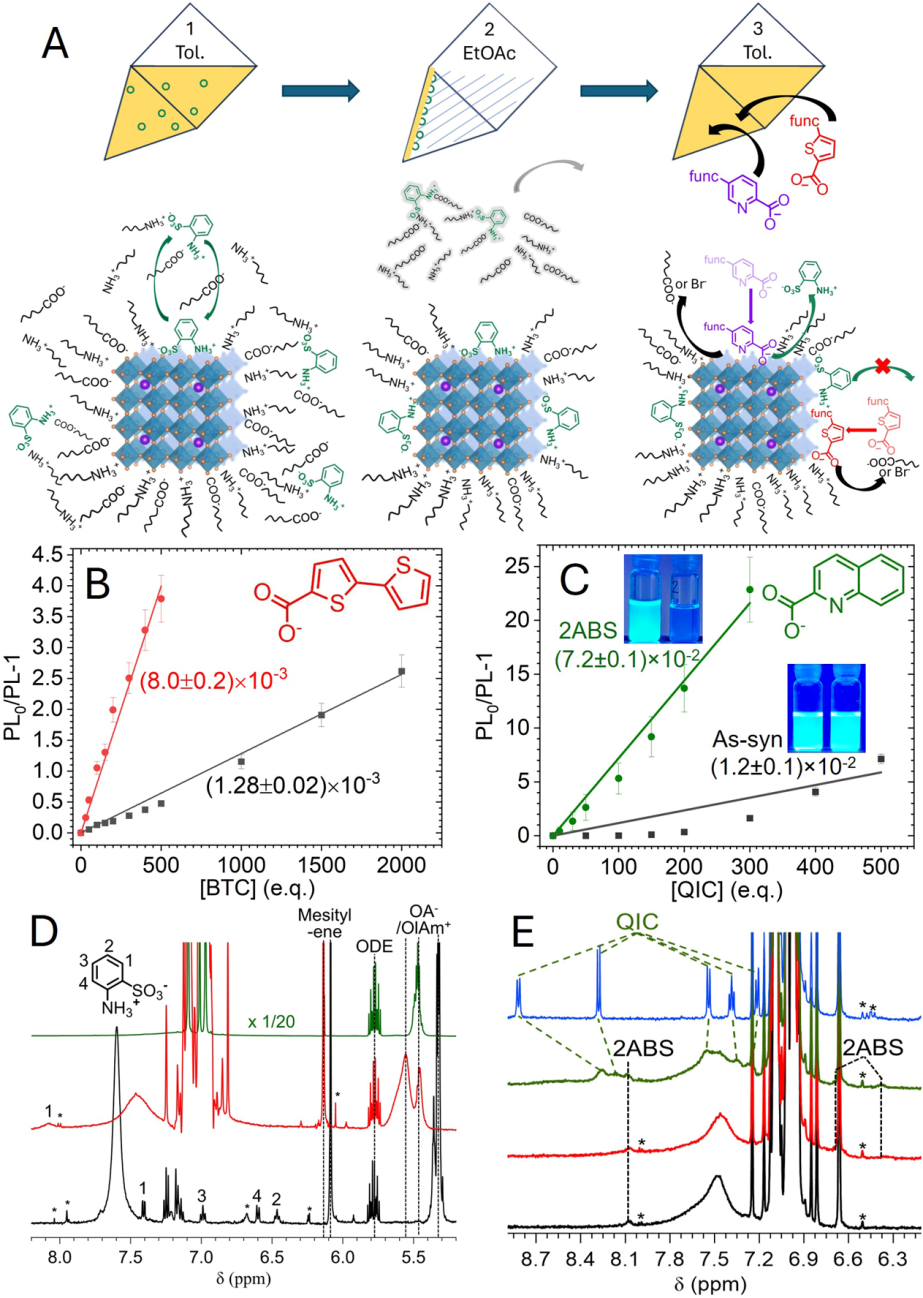
(A) Schematic illustration of the surface functionalization procedure. (1) 2ABS-protection: To 1 mL of 5 μM PNC solution in toluene, 2.5 μmol 2ABS solid was added, and the mixture was stirred overnight, after which toluene was removed by evaporation to obtain 2ABS-protected PNCs. (2) Purification: 2ABS-protected PNCs were washed with 4 mL anti-solvent, ethyl acetate, and the suspension was centrifuged at 3500 rpm, 0 ^o^C. The upper solution containing the unbound synthetic and 2ABS ligands was discarded, and 2ABS-purified PNCs were obtained in the precipitate. (3) Functionalization with acceptors: 2ABS-purified PNCs were redissolved in a non-polar solvent, such as toluene. Functional molecules, such as acceptors, were titrated to the solution and attached to the PNC surface via chelating anchoring groups. (B) and (C) PL quenching plots with BTC and QIC for as-synthesized (black) and 2ABS-purified PNCs (red with BTC and green with QIC). The straight lines represent the linear fit of *PL*_0_ */PL*-1 against the equivalent of the quenchers to indicate the trend. Inset: photos of as-synthesized (lower) and 2ABS-purified (upper) PNC samples under 365 nm UV illumination before (left) and after (right) adding 200 e.q. of QIC. (D) ^1^H-NMR absorption of the PNC samples in toluene-D8 before (green) and after (red) 2ABS-purification. The spectrum before 2ABS treatment was scaled by 1/20. We also degraded the purified PNC sample in DMSO–D6 (black) to release surface-bound 2ABS for quantifying 2ABS. (E) ^1^H-NMR spectra of PNC samples with the addition of the model acceptors. From bottom to top: 2ABS-purified PNCs, 2ABS-purified PNCs + 100 e.q. BTC, 2ABS-purified PNCs + 100 e.q. QIC, and as-synthesized PNCs + 100 e.q. QIC. NMR bands belong to QIC, and 2ABS are noted with green dashed and black dotted lines. NMR Spectra were normalized to the integrated intensity of the mesitylene internal standard. *denoted signals of impurity.

We then relied on the chelating coordination of the heterocyclic carboxylate structures to attach acceptor motifs to the PNC surface. UV–vis absorption of samples after applying BTC and QIC showed no significant changes (Figure S1). Our previous study identified that picolinate (PIC) and 2-thiophene-carboxylate (TC) can effectively passivate the surface defects for their strong affinities to the PNC surface.^28^ These chelating ligands, with large HOMO-LUMO gaps, form type I junctions with the PNC core when attached to the surface, which effectively reduce the exciton trapping and enhance photoluminescence (PL) of 2ABS-purified PNCs (Figure S2). We extended the conjugated systems of the prototype ligands to quinaldate (QIC) and bithiophene carboxylate (BTC), and observed effective quenching of the PNC’s PL intensity, as shown in Figure S3 and summarized in Figure 1B,C. Given that PIC and TC served as passivation ligands, their derivatives, QIC and BTC, which had similar coordination to the PNC surface, should not introduce more surface defects when bound to the surface. Therefore, the quenching of the PL must be due to either energy transfer or charge carrier transfer to the bound acceptors, as we will discuss in detail later. QIC demonstrated a quenching capability about an order of magnitude greater than BTC for both as-synthesized and 2ABS-purified PNCs. Prominently, when we titrated quenchers to the PNC samples in the same manner and allowed them to interact for the same contact time, the 2ABS-purified PNC demonstrated a 6-fold increase of quenching constants compared to the as-synthesized sample. The stark contrast of the quenching behavior for samples before and after 2ABS-purification is illustrated in Figure 1B,C.

We attributed the enhanced quenching effect of the purified PNC to the improved surface coupling of the acceptors due to the decline of competitive adsorption after reconstructing the surface chemical environment through purification. ^1^H-NMR spectra shown in Figure 1D indicated changes in the adsorption status of different ligands before and after the 2ABS purification treatment. 96% of the original synthetic ligands were removed, leaving 352 ± 33 oleylammonium (OlAm^+^) and oleate (OA^–^) for each PNC, and the original 3∼5000 e.q. per PNCs, synthetic solvent, ODE, was reduced to about 50 e.q. after purification. The alkene resonance of OlAm^+^/OA^–^ split into two bands after purification: a broadened and downfield shifted band centered at 5.55 ppm, accounting for 86% of the total integrated intensity, and a smaller narrow band (14%) remaining at the same peak position as the as-synthesized sample (5.47 ppm). The former belonged to the remaining 303 e.q. OlAm^+^/Br^–^, which had a relatively strong association with the PNC surface, while the latter belonged to 49 e.q. weakly bound OA^–^ ligands.^24,28^ In toluene, 2ABS was firmly attached to the PNC surface, as shown by the drastically broadened ^1^H-NMR signals: only one broad band at 8.07 ppm could be identified, and the rest of its proton resonance was too broad to be observed.^32,43,44^ The number of 2ABS per PNC was quantified as 44 ± 10 after we decomposed the purified PNC sample in DMSO–D6 to release surface ligands, suggesting that the strongly bound 2ABS only occupied 13% surface Pb (II) sites, over the totally 338 sites of the PNC sample (*d* = 4.4 nm) used in the present study. The majority of surface sites (338 × 87% = 294) were dynamically covered by ∼350 synthetic ligands, OlAm^+^/Br^–^, and OA^–^. The greatly lowered concentration of synthetic ligands in the 2ABS-purified PNC compared to the as-synthesized sample made its surface more accessible for functionalization. Acceptors with a chelating anchoring motif could easily replace the synthetic ligands to achieve firm anchoring, given that the binding affinity of OlAm^+^/PIC^–^ is 15- to 30-fold greater than OlAm^+^/Br^–^.^28^

^1^HNMR spectra shown in Figure 1E demonstrated the binding of BTC and QIC quenchers on the reconstructed PNC surface. In contrast to the sharp proton resonance peaks observed when applying the quencher to the as-synthesized sample, NMR bands were significantly broadened in the presence of the purified sample, indicating the strengthening of surface associations for the quenchers. We also noticed the enhanced proton resonance signals of 2ABS with the addition of quenchers. When QIC was added, the ^1^H­(1) band at 8.07 ppm grew up, and two new bands at 6.38 and 6.66 ppm belonging to ^1^H­(2) and ^1^H­(4) of 2ABS emerged, implying that QIC could replace 2ABS on the PNC surface. BTC signals were not directly observable in the ^1^HNMR spectrum due to the overlap with the solvent resonance, but its binding could still be inferred from the appearance of the 2ABS proton resonance at 6.38 pm. The rise of 2ABS ^1^H-NMR signals when adding BTC to the PNC sample was less prominent compared to the case of QIC, suggesting the relative binding affinity of BTC is likely weaker than the latter. The N-heterocyclic-carboxylate structure is more suitable for anchoring functional groups to the PNC surface compared to the thiophene-carboxylate. This two-step functionalization method also works for PNCs with different sizes, as we demonstrated for a larger PNC with bandgap absorption peaked at 484 nm (Figure S4A). We rationalized three crucial advantages of this 2ABS-assisted functionalization procedure. (1) The purification step greatly reduced competitive adsorption from the excess number of synthetic ligands in the as-synthesized sample while preserving the integrity of the nanocrystal. (2) 2ABS-purification only introduced a limited number of protective ligands, keeping most surface sites covered by weakly bound synthetic ligands and open to functionalization. (3) 2ABS has a moderate binding affinity compared to the chelating anchoring group, such as N-heterocyclic-carboxylate, and could be replaced to further increase the amount of tethered functional groups.

### Energy and Charge Carrier Transfer Mechanisms from PNC to the Model Acceptors

We employ transient absorption (TA) spectroscopy to unravel the different mechanisms of PL quenching for BTC and QIC acceptors on the PNC surface (Figure 2). We first focus on the kinetics of the ground state bleach (GSB) signal of PNCs at 468 nm (Figure 2D). The GSB signal is contributed by both electron and hole-filling effects at the conduction and valence band edges,^45^ which reveals the evolution of the photoinduced carriers in different systems. After the purification step, the kinetics GSB signal of PNCs must be fit with three exponential components (Figure S5 and Table 1), including one component with a time constant of 25 ps. This ultrafast decay component was assigned to the fast trapping of the photoinduced carrier to the deep trap caused by surface defects.^42^ Decorating the PNC surface with chelating ligands removed the ultrafast exciton trapping process, and the kinetic traces could be fit with a two-step exponential decay (Table 1). For PIC and TC-passivated samples (Figure S5), the initial decay component had a time constant of 300-700 ps, and was assigned to the exciton trapping by intrinsic defects that cannot be passivated by surface ligands.^28,46^ The majority part of the GSB decay reflected decay component had time constants of about 5 ns, which agreed well with the time-resolved photoluminescence lifetimes (Figure S6), and were consistent with the reported radiative recombination lifetime in CsPbBr_3_ PNCs.^25,26^ The BTC and QIC decoration suppressed the ultrafast exciton trapping process at surface defects similarly as the generic ligands, i.e., TC and PIC, excluding the possibility that PL quenching observed for BTC and QIC was caused by surface defects induced during functionalization. In contrast to the elongated excitation lifetimes in the passivated samples, both BTC and QIC prominently accelerated the overall decay rates of the GSB kinetics, indicating the removal of photoinduced charge carriers through pathways that competed with intrinsic radiative recombination.

**Figure 2:**
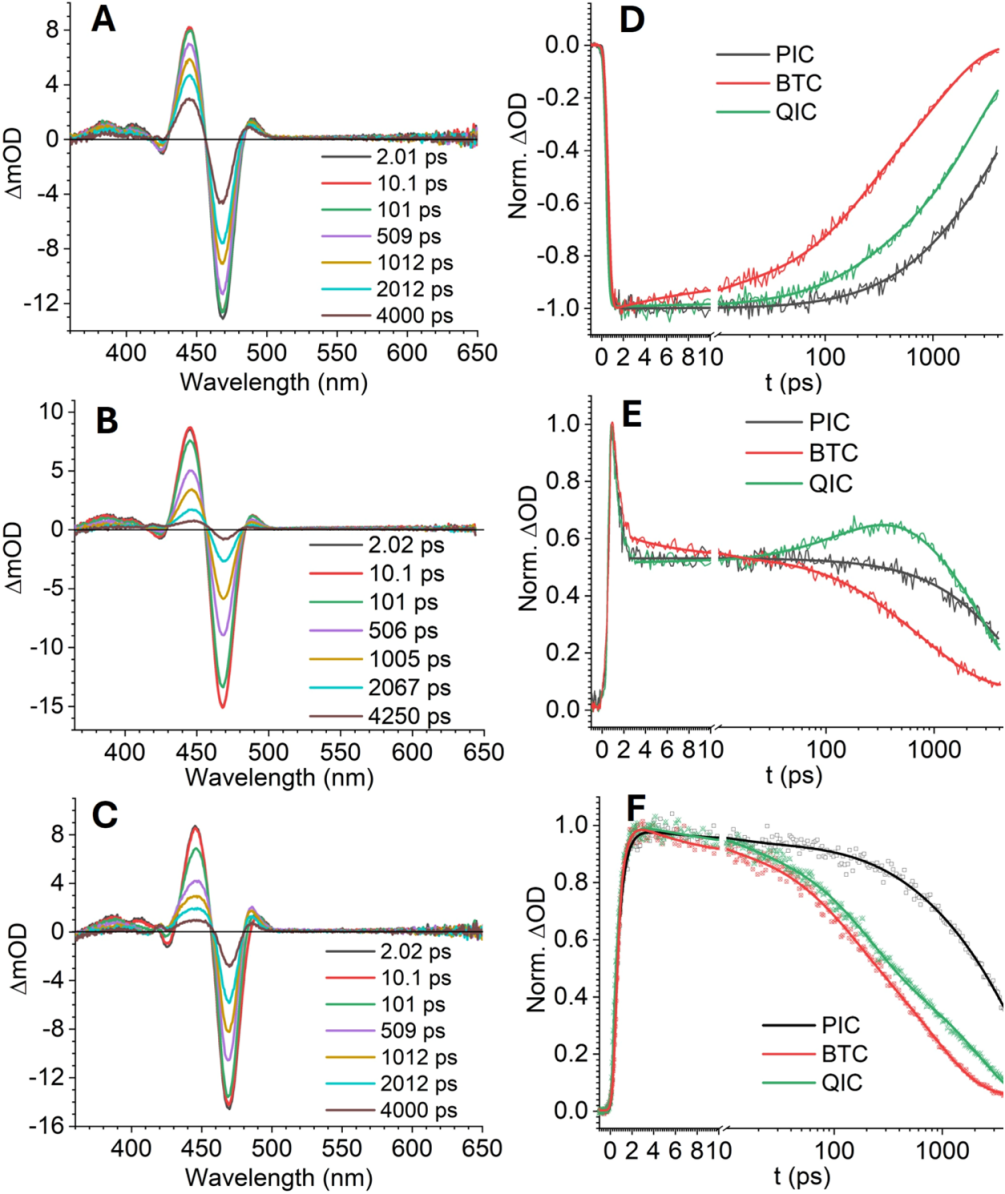
Sub-ps-to-ns transient absorption spectroscopy for PNC samples. TA spectra at selective time delays for 0.80 μM PNC in toluene with (A)­150 PIC, (B) 500 e.q. BTC, and (C) 200 e.q. QIC. The variation of TA signals for different PNC samples is due to the change of the pump focus. TA kinetics of samples in (A) to (C) monitored at (D) the GSB minimum signal at 468 nm, (E) the PIA signals at 485 nm, and (F) the PIA signals at 445 nm. The TC-passivated sample shows a similar evolution as the PIC-passivated ones, and the data are illustrated in Figure S5. Kinetic traces are fitted with multiple exponential decay components, and the fitting parameters are listed in Table 1.

**Table 1:** Photoluminescence Quantum Yields/Lifetimes and Multiexponential Fitting Parameters of TA Kinetics in Figure 2 for Different PNC Samples

			TA				
surface	Φ_PL_	τ_TRPL_ (ns)	signal (nm)	τ_1_ (ps) A_1_	τ_2_ (ps) A_2_	τ_3_ (ns) A_3_	τ_ave_ (ns)^a^
2ABS-purified	0.61–0.89	4.82 ± 0.02	GSB	25 ± 9	390 ± 90	4.9 ± 0.3	3.9
			468	0.05 ± 0.01	0.17 ± 0.02	0.78 ± 0.02	
PIC-passivated	>0.92	5.06 ± 0.02	GSB		700 ± 300	5.1 ± 0.6	4.6
			468		0.12 ± 0.06	0.88 ± 0.06	
			PIA^b^	8 ± 3	500 ± 100	5.4 ± 0.2	
			445	0.06 ± 0.01	0.10 ± 0.02	0.84 ± 0.02	
			PIA^c^			5.7 ± 0.1	
			485				
200 e.q. QIC	0.064 ± 0.005	<IRF	GSB		170 ± 20	2.53 ± 0.06	2.2
			468		0.16 ± 0.01	0.84 ± 0.01	
			PIA	11 ± 3	182 ± 9	2.34 ± 0.07	
			445	0.08 ± 0.01	0.43 ± 0.01	0.49 ± 0.01	
			PIA		190 ± 20	2.8 ± 0.3	
			485		(decay)	(growth)	
TC-passivated	>0.92	5.52 ± 0.03	GSB		300 ± 30	4.9 ± 0.1	4.5
			468		0.92 ± 0.01	0.08 ± 0.01	
			PIA	5 ± 2	250 ± 20	5.2 ± 0.2	
			445	0.03 ± 0.01	0.11 ± 0.01	0.85 ± 01	
			PIA			5.5 ± 0.7	
			485				
500 e.q. BTC	0.22 ± 0.02	<IRF	GSB		160 ± 20	1.06 ± 0.04	0.99
			468		0.92 ± 0.02	0.08 ± 0.02	
			PIA^d^	2.4 ± 0.9	180 ± 30	1.05 ± 0.04	
			445	0.08 ± 0.03	0.12 ± 0.03	0.74 ± 0.02	
			PIA			1.2 ± 0.3	
			485			0.62 ± 0.11	

a Averaged by the percentage of amplitudes of different exponential components.

b For all PIA 455 nm signals, the initial growth component with a time constant of ∼0.6 ps was not listed.

c For all PIA 485 nm signals, the initial fast decay with a time constant of ∼0.6 ps was not listed.

d A persistent component with 6% of the total amplitude was required for fitting the kinetic trace, which might correspond to the absorption of the TEnT product.

For the BTC-decorated sample, we observed that the GSB decayed with an average lifetime of 0.99 ns, in which 92% of the amplitude decayed with a time constant of 1.06 ± 0.04 ns. Assuming that the intrinsic exciton decay lifetime was the same as the TC-passivated sample (4.5 ns), we calculated that BTC quenched the PNC exciton with a decay rate constant, $k_{q}^{\mathrm{BTC}}=\frac{1}{0.99}-\frac{1}{4.5}=0.79$ ns^–1^. This value closely agreed with the quenching rate constant (0.89 ns^–1^) obtained from the Stern–Volmer analysis shown in Figure 1B. The concerted GSB decay and the PL quenching kinetics suggested an energy transfer mechanism where the BTC acceptor extracted the photoinduced exciton, both the electron and hole, simultaneously from the donor PNC.^47^

Analyzing the relative energy level alignments of the valence and conduction bands of PNCs and the BTC HOMO/LUMO suggested triplet energy transfer (TEnT) as the quenching mechanism. The relative energy levels in the PNC-BTC complex do not favor single carrier transfer processes, such as electron transfer or hole transfer from PNC to BTC,^48^ as shown in Figure 3A. Given that the optically allowed transition of BTC has energy (337 nm, 3.68 eV) that is much higher than the lowest excitonic state of PNCs (2.66 eV) used in this research, we can exclude the possibility of Förster resonance energy transfer. The T_1_ state energy of BTC was determined between 2.2 and 2.3 eV,^49–51^ making the energy transfer pathway to the triplet excited state accessible. The strong spin-orbit coupling within the CsPbBr_3_ PNC makes the exciton poorly defined in terms of spin multiplicity and favors the triple–triplet energy transfer (TEnT).^20^ Though the charge-separated states are unavailable in the PNC-BTC complex, they may still mediate the triplet energy transfer processes.^52^ Based on the energy transfer rate constant of 0.79 ns^–1^, the yield of triplet energy transfer, Φ_TEnT_ = *k*_TEnT_ τ = 0.80. Our TA experiment did not capture signals that could be unambiguously assigned to the triplet state of BTC. The T_1_ state of bithiophene has an absorption peak at 400 nm,^53^ which is difficult to distinguish from the TA spectra shown in Figure 2B due to the relatively low absorptivity (2 × 10^4^ M^–1^ cm^–1^) and the overlap with the highly absorptive PNC first-excitonic band (2 × 10^6^ M^–1^ cm^–1^). Triplet energy transfer from semiconductor nanocrystals to thiophene derivatives has been observed,^54^ and we report here the example of efficient generation of the triplet excited state of the thiophene-based organic semiconductor through TEnT from PNCs.

**Figure 3:**
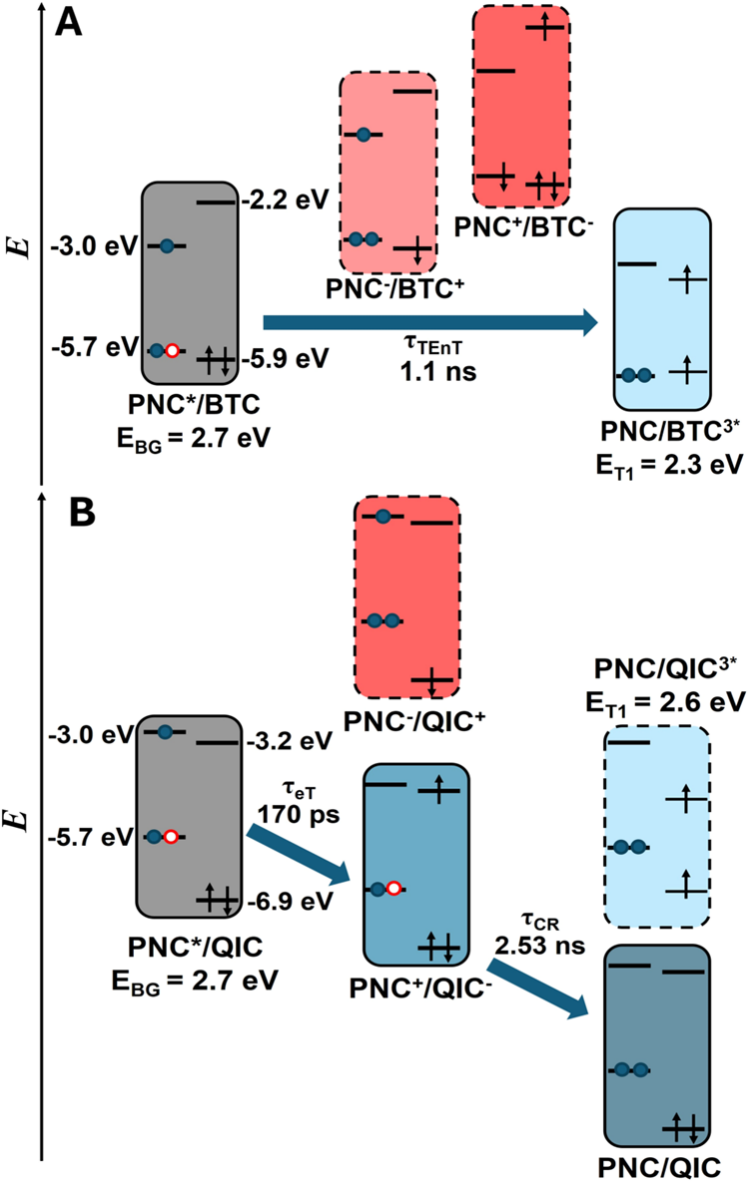
Energy diagram of (A) triplet–triplet energy transfer in the PNC-BTC complex and (B) electron transfer/charge recombination in the PNC-QIC complex and molecular acceptors. Blue and red shaded boxes represent energetically favorable and unfavorable states, respectively. Unobserved states were circled with dashed lines.

The biexponential fit for the kinetics of the GSB signal of the QIC-decorated sample revealed a fast process with a time constant of 170 ± 20 ps that accounted for 16% of the GSB amplitude and a slow decay with a time constant of 2.53 ± 0.06 ns that accounted for the rest 84% of the GSB amplitude. It was noticeable that the quenching effect induced by 200 e.q. QIC was almost 4-fold more substantial than 500 e.q. BTC in Figure 1, but the average lifetime of the GSB signal in the PNC-QIC complex was 2.2 ns, remarkably longer than that of the BTC-decorated sample. The distinct kinetic features implied a different PL quenching mechanism for QIC. From the Stern–Volmer analysis in Figure 1C, we determined the PL quenching rate constant of 200 e.q. QIC is 3.1 ns^–1^, corresponding to a PNC exciton lifetime of 300 ps. This lifetime was 7-fold shorter than the average GSB decay lifetime but was on the same order of magnitude as the time constant of the fast decay component in the biexponential fit. We, therefore, suggested that the rapid sub-nanosecond decay process observed in the GSB decay was responsible for PL quenching. The two-step decay in the GSB kinetics should be assigned to two sequential processes: i.e., (1) the PNC underwent fast photoinduced charge carrier transfer (CT) to the QIC acceptor in 170-300 ps, which resulted in drastic PL quenching, and (2) the separated electron and hole in the PNC-QIC complex recombined in about 2.5 ns, as reflected by the slow decay component in the GSB kinetics.

The energy level alignment in the PNC-QIC complex suggested that the initial CT process is electron transfer (eT), as illustrated in Figure 3B. The LUMO energy of QIC (−3.2 eV *vs*. vacuum), as determined by cyclic voltammetry (Figure S7), lies below the conduction band edge of the PNC (E_CBM_ = −3.0∼−2.8 eV *vs*. vacuum),^32,52^ providing sufficient driving force for eT. Direct hole transfer (hT) from the valence band (E_VBM_ = −5.7 eV *vs*. vacuum) to the QIC LUMO (−6.9 eV *vs*. vacuum) is unfavorable. The T_1_ state energy of QIC was determined as 2.6 eV according to the phosphorescence spectrum measured at 77K (Figure S8), making it also energetically accessible for the bandedge exciton of the PNC (E_BG_ = 2.66 eV). However, electron transfer can kinetically outcompete triplet energy transfer for its less dependence on donor–acceptor wavefunction overlap.^55^ Recombination of the charge-separated state on the semiconductor nanocrystal surface can generate either the T_1_ or the S_0_ state of organic acceptors.^56–59^ We tried to identify the T_1_ state of QIC by measuring the PL of the QIC-decorated PNC at 77K with 440 nm excitation, when only the PNC was excited, but did not observe the phosphorescence from QIC (Figure S8). Therefore, we conclude here that charge recombination (CR) directly brings the PNC-QIC system back to the ground state through a thermodynamically downhill pathway.

### Interpretation of TA Spectral/Kinetic Features of PNCs in Charge Carrier Transfer Processes

We have identified the triplet energy transfer and single charge carrier transfer processes in different PNC-acceptor complexes based on comparing TA and PL quenching kinetics. However, other spectral and kinetic features in TA spectroscopy also provide detailed information about these processes and deserve a close review.

First, the evolution of the photoinduced absorption (PIA) signal on the lower energy side of the GSB reflected the photoinduced charge separation process in the PNC-QIC system (Figure 2E). For all three samples shown in Figure 2, this PIA signal consisted of a rapid initial decay with a lifetime of ∼0.6 ps, followed by relatively slow evolution with persistent amplitude until the late time delay. The sub-ps evolution of the PIA had been assigned to a biexciton feature following the assignment for the TA feature of metal chalcogenide nanocrystals,^60,61^ but was then associated with the polaron formation process.^62–65^ This sub-ps PIA development is, anyway, irrelevant to the time-scale of energy and charge carrier transfer processes discussed in the present study. The slowly evolving kinetic component of the PIA had two origins: (1) the localized hole state as suggested by previous research in strongly quantum-confined perovskite systems,^66,67^ and (2) the redshifted PNC bandedge transition due to the Stark effect induced by charge-separation at the nanoparticle/ligand interface.^68–70^ In the passivated and BTC-decorated samples, the slowly decaying PIA signal should be assigned to the localized hole state, and its decay was concerted to the evolution of the GSB signal. The PNC-QIC complex differed from the former by showing prominent growth after the initial ultrafast evolution, and the growth time constant (190 ± 20 ps) was coincident with the early stage of the GSB decay that was assigned to eT (170 ± 20 ps). We assigned this PIA growth to the Stark effect induced by the formation of the interfacial charge-separated state due to photoinduced eT from PNC to the surface-adsorbed QIC. The PIA signal then decayed with a time constant of 2.8 ± 0.1 ns, following the same pace as the CR component in the GSB signal evolution (2.53 ± 0.06 ns), due to the elimination of the charge-separated state. Therefore, this lower energy PIA signal is a signature of charge carrier transfer across the nanocrystal surface, and its evolution can be used to track the CT/CR kinetics.

The kinetics of the PIA signal on the high-energy side of the GSB also reflected the evolution of photoinduced carriers (Figure 2F). This blue PIA signal originated from symmetry-forbidden transitions that were activated by the formation of polarons in small-sized PNCs, and the rise of this signal coincided with the initial ultrafast decay of the PIA on the lower energy side of the GSB.^65^ The decay of the 445 nm PIA could be interpreted as the transition allowed by polaron-induced lattice distortion diminished and eventually became forbidden with the elimination of photoinduced carriers. For both the passivated and BTC-capped PNCs, the 445 nm PIA decayed at a pace that closely followed the GSB decay, as illustrated in Figure 4, which was interpreted as the simultaneous vanishing of the photoinduced electron and hole through either the radiative recombination or the energy transfer pathways. A slight discrepancy between the 455 nm PIA and the GSB kinetics was noticed at the early time kinetics, where the PIA signal showed an extra small decay component with a time constant of a few picoseconds (Table 2). We attributed this decay to the carrier-trapping process.

**Figure 4:**
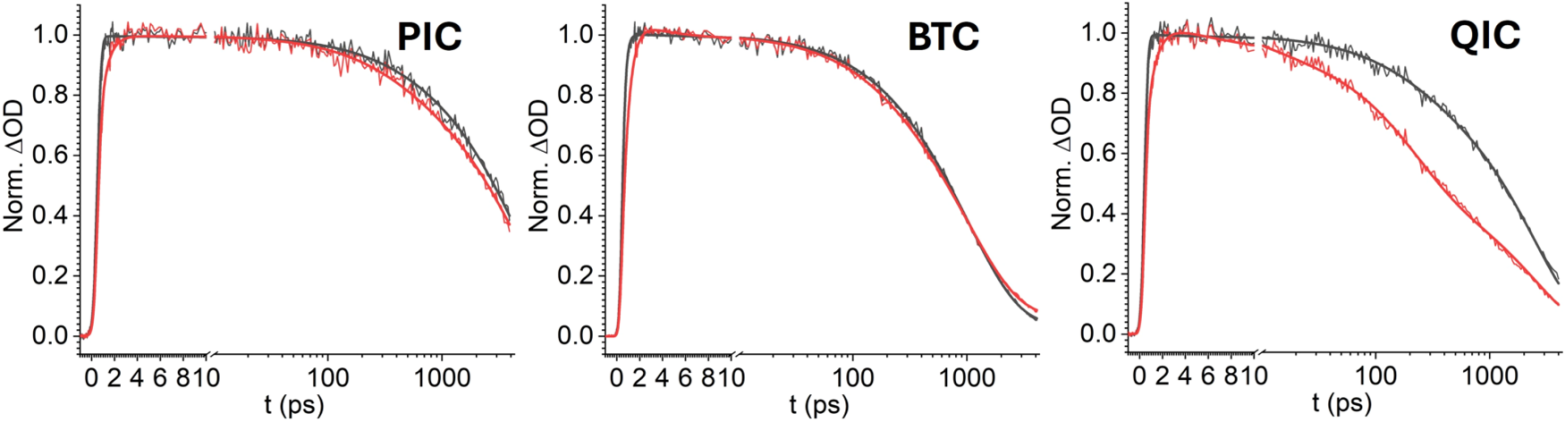
Kinetic comparison of the GSB signal at 468 nm (black) and the PIA signal at 445 nm (red) for 2ABS-treated PNC samples after applying 200 e.q. PIC, 500 e.q. BTC and 200 e.q. QIC. Global fitting with shared decay time constants was applied, and the fitting parameters are listed in Table 2.

**Table 2:** Global Fitting Results with Shared Time Constants (τ_2_ and τ_3_)

ligand	signal (nm)	τ_r_ (ps)^a^	τ_1_ (ps) A_1_	τ_2_ (ps), A_2_	τ_3_ (ns), A_3_
PIC	GSB			390 ± 60	4.7 ± 0.2
	468			0.06 ± 0.01	0.94 ± 0.01
	PIA	0.48 ± 0.03		390 ± 60	4.7 ± 0.2
	445			0.13 ± 0.02	0.87 ± 0.02
BTC	GSB			190 ± 30	1.08 ± 0.03
	468			0.08 ± 0.02	0.88 ± 0.01
	PIA	0.59 ± 0.04	2.3 ± 0.9	190 ± 30	1.08 ± 0.03
	445		0.09 ± 0.05	0.12 ± 0.02	0.74 ± 0.02
QIC	GSB			199 ± 9	2.48 ± 0.05
	468			0.15 ± 0.01	0.85 ± 0.01
	PIA	0.52 ± 0.02	9 ± 3	199 ± 9	2.48 ± 0.05
	445		0.09 ± 0.01	0.49 ± 0.01	0.42 ± 0.02

a An exponential growth component is needed to fit the rise of the PIA-445 signal.

The PNC-QIC complex was distinct from the other two systems shown in Figure 4 by showing a prominent discrepancy between the kinetics of the 445 nm PIA and the GSB. However, a close comparison of the kinetic fits of the two traces revealed connections: except for the <10 ps decay component that only accounted for 8% of the PIA amplitude, the time constants of the two major decay steps of the 445 nm PIA coincided with those obtained from the biexponential fitting of the GSB signal (182 ± 9 ps *vs*. 170 ± 20 ps, and 2.34 ± 0.07 ns *vs*. 2.53 ± 0.06 ns, respectively, as listed in Table 1). In fact, the two kinetic traces (468 nm GSB and 445 nm PIA) could be globally fit with shared time constants, as shown in Figure 4, implying that the two-stage decay of the 445 nm PIA originated from the same carrier evolution processes as the GSB. The prominent difference between the 445 nm PIA and the GSB kinetic traces of the PNC-QIC complex lay in the relative decay amplitudes that corresponded to the eT and the hole removal CR processes, respectively: the two processes contributed roughly equal amplitudes in the 445 nm PIA (49 vs. 42%), whereas the contribution of the eT stage was much smaller than the hole removal in the GSB evolution.

In the GSB kinetics of the PNC-QIC complex, eT only accounted for 16% of the GSB decay, whereas the remaining 84% of the GSB amplitude was attributed to hole removal through charge recombination. This large discrepancy between the electron and hole amplitudes in the GSB signal is abnormal, given that both the valence and conduction band edges of PNC have a 2-fold degeneracy,^71^ and the electron and the hole have similar effective masses.^72^ In fact, it has been determined that for CsPbBr_3_ PNC with an edge size of ∼10 nm, the electron and hole-filling effects contribute 67.2 and 32.8% in the GSB, respectively.^45^ We speculate that our abnormal observation reflects the different influences of the polaron-induced lattice distortion on the originally allowed bandedge transition. Zhu and coauthors predicted that the injection of positively and negatively charged carriers has different effects on the perovskite lattice: the former draws the Cs^+^ cation to the cubic sites, resulting in a structure with higher symmetry, while the latter causes the opposite lattice change.^62^ For small PNCs in the strongly quantum-confined regime, the disturbance of the lattice symmetry can allow the originally forbidden transitions, as seen in the appearance of the PIA at 445 nm.^65^ It is plausible that the same lattice distortion alters the oscillator strength of originally allowed bandedge transitions. During the TA experiment, the eT process that removed negatively charged carriers caused lattice relaxation that enlarged the oscillator strength of the remaining bandedge transition and, hence, offset the corresponding GSB recovery. This effect might not be prominent in bulk perovskite or large-sized PNCs (i.e., ∼10 nm) due to the limited lattice region affected by the polaron, but becomes significant in small-sized PNCs. In fact, when measuring the eT/CR process in a donor/acceptor system consisting of larger PNCs (5.5 nm), we found the contributions of electron and hole-filling to the GSB (at 484 nm) changed to 40 and 60%, respectively (Figure S4B,C, and Table S1), which are closer to those in the bulk-like PNC. This observation suggested that the polaron-affected TA spectral feature evolves with the increase of the PNC size. The polaron-affected transitions can also explain the slight discrepancies in the early time kinetics of the passivated and BTC-decorated PNCs shown in Figure 4: the small amplitude, ultrafast decay observed in the PIA-445 nm signals likely corresponded to the remaining electron trapping process that caused similar lattice change as the electron transfer, while the same effect only induced small recovery in the GSB kinetics that was not captured by kinetic fitting. This notable kinetic signature can be employed to distinguish electron transfer from other transfer processes in strongly quantum-confined PNCs.

## Conclusions

Using appropriate protective ligands, we reconstructed the surface chemical environment of as-synthesized PNCs to facilitate the attachment of energy and charge carrier acceptors. PNCs with improved surface accessibility to molecular acceptors can achieve more efficient exciton and carrier transportation, which will promote the performance of PNCs as photosensitizers and photocatalysts. Chelating ligands, particularly those with a picolinate motif, are stable anchoring groups for grafting functional ligands. It is reasonable to believe that this surface functionalization strategy will benefit the development of PNC-based optoelectronics by promoting mutual carrier migration across the ligand layer. We demonstrated an efficient triplet energy transfer process from PNCs to bithiophene acceptors, and observed an electron transfer/charge recombination process between PNCs and quinoline acceptors using transient absorption spectroscopy. We identified two characteristic TA spectral evolutions associated with the CT process in strongly quantum-confined PNCs. (1) The evolution of the Stark-effect-induced PIA at the lower energy side of the bandedge absorption closely reflects the kinetics of the charge separation and recombination processes. (2) A comparison of relative amplitudes of the kinetic components in the GSB and the PIA at the higher energy side of bandedge adsorption indicates the polaron effect during photoinduced electron transfer. These spectral and kinetic features provide systematic evidence for distinguishing charge transfer and energy transfer processes. The present study once again demonstrates the strong influence of surface chemistry on the energy and charge transfer processes from PNCs. Starting from the presented surface modification method, our group is working on systematically tuning the chemical components of the PNC ligand shell to modulate the transfer kinetics. We believe that with tunable surface environments, we will be able to decipher the crucial factors of surface chemistry that determine the photophysical/chemical mechanism of PNCs.

## Experimental Section

### Materials

The chemicals listed were used as received unless stated otherwise: hexanes (Certified ACS, Fisher), toluene (Certified ACS, Fisher), and acetone (Certified ACS, Fisher). The above-mentioned solvents were dried with the molecular sieves (4 Å) overnight before use. Ethyl acetate (Certified ACS, Fisher), oleic acid (OA, 90%, Sigma-Aldrich), oleylamine (OlAm, 70%, Sigma-Aldrich), 1-octadecene (ODE, Tech 90%, Fisher), lead­(II) bromide (PbBr_2_, 99.998, Alfa-Aesar), anhydrous zinc bromide (ZnBr_2_, 99.9%, Alfa-Aesar), cesium carbonate (Cs_2_CO_3_, 99.9%, Acros), 2-ammonium benzenesulfonate (2ABS, 98%, TCI), Quinaldic acid (QIC, 98.0%, TCI), 2,2-Bithiophene-5-carboxylic acid (BTCA, 97%, Thermo Scientific), toluene-d_8_ (Tol-D8, 99.5% D, Cambridge Isotope Lab), and dimethyl sulfoxide-d_6_ (DMSO-D6, 99.9% D, Cambridge Isotope Lab).

### Synthesis of CsPbBr_3_ Perovskite Nanocrystals

A modified procedure, adapted from ref 73, was used to synthesize the PNCs as follows. The ligands, the Cs precursor, and the Pb precursor were prepared separately in three flasks. To prepare the cesium oleate precursor, 250 mg of CsCO_3_ was dissolved in 9 mL of ODE with 0.9 mL of OA. The solution was dried under a vacuum at 120 °C for 1 h, then after switching to an N_2_ atmosphere, the temperature was raised to 150 °C, and maintained for 10 min. The solution temperature was lowered to 100°C and was kept under N_2_ atmosphere before use. The ligands (OA and OlAm) (1:1 vol) were dried under vacuum at 120° for 1 h. The reaction flask was loaded with 10 mL of ODE, 150 mg of PbBr_2_, and 368 mg of ZnBr_2_. The mixture was dried under a vacuum at 120 °C for an hour. Then, 9.5 mL of the mixing ligands were added to the flask to dissolve all salts. The reaction mixture was then set to 100 °C when 0.8 mL of the CsOA precursor was injected. The reaction was allowed to proceed for 60 s before being rapidly cooled with an ice bath. The undissolved solids were removed by centrifugation at 3500 rpm for 45 min. The upper solution was stored overnight in a dry desiccator, allowing the undesired various products to precipitate out of the solution. PNCs with the desired size were precipitated using acetone and recovered by 5 min centrifugation. The final product was dried under vacuum overnight and redissolved in hexanes to stock.

### Steady-State Optical Spectroscopy

All UV-Vis measurements were performed with an Agilent Cary 5000 spectrometer using a quartz cuvette with a path length of 4 mm. The photoluminescence spectra of the PNC samples were measured with a Horiba FluoroMax 3 fluorometer with an excitation wavelength of 412 nm. The PLQYs were determined by comparing the integrated intensities of the samples to that of a fluorescence reference standard, 9,10-diphenyl anthracene.

### Nuclear Magnetic Resonance (NMR) Spectroscopy

All NMR measurements were performed with a 500 MHz Bruker spectrometer equipped with a reversed probe to enhance the ^1^H sensitivity. 1D ^1^H-NMR was acquired with a standard pulse sequence, and the relaxation delay was 7 s. Mesitylene or tri-methoxy benzene was used as internal standards (typically 1 mM) in NMR measurements for quantitative analysis.

### Transient Absorption Spectroscopy

Details about the TA system can be found in ref.^32^ Samples were pumped with polarization-scrambled, 420 nm pulses that were chopped to 500 Hz. The pump beam was softly focused on the sample and attenuated to 40 nJ per pulse. The supercontinuum white light probe is generated using a 2 mm thick CaF_**2**_ crystal and covers a spectral window from 350 to 700 nm. This optical delay setup provided a time window of up to 4200 ps. The white light was divided into two arms by a beamsplitter, with a 70% reflective beam to probe the sample and the rest of the beam transmitted to serve as the reference. Both the sample and the reference beams were focused on the sample plane but separated vertically by ∼2 cm. The pump and the sample beam overlapped at the sample. The diverged sample and reference beams were collimated and focused to the slit of an Acton spectrometer (SpectralPro-300i) and separately dispersed to two vertically aligned photodiode arrays (1024 pixels, EB Stresing) by a 150 gr/mm grating. TA signals were calculated pixel-by-pixel using the formula $\Delta OD=-\log\Delta OD=-\log({\left(\frac{I_{S}}{I_{R}}\right)}_{pump-on}/{\left(\frac{I_{R}}{I_{S}}\right)}_{pump-off})$ , where *I*_S_ and *I*_R_ were the intensities of the sample and reference, respectively.

## Supplementary Material



## Notes

Characterization of PNCs during functionalization; passivation effect of PNC with PIC and TC; PL quenching with QIC and BTC; functionalization for large PNCs; TA for PIC and TC-passivated PNCs; TRPL; energy levels of QIC (PDF)
