# Supplementary material for: Facilitating Energy and Charge Transfer from CsPbBr3 Perovskite Nanocrystals via Ligand Shell Reconstruction
Source: ACS Appl Mater Interfaces. 2025 May 15;17(21):31237–47. doi: 10.1021/acsami.5c03095 (PMC12123618; doi:10.1021/acsami.5c03095)
Supplement: Supplementary file 1 [file am5c03095_si_001.pdf]

Supporting Information

Facilitating Energy and Charge Transfer from $CsPbBr_3$ Perovskite Nanocrystals via Ligand Shell Reconstruction

Authors: Aaron Malinoski, Jingheng Yuan, Chen Wang*

Department of Chemistry and Biochemistry, Queens College, CUNY, Flushing, NY 11367, USA
The Graduate Center of CUNY, New York, NY 10016, USA

**corresponding author. Email: chen.wang@qc.cuny.edu*

Table of Contents **Page**

1. Characterization of 2ABS-purified and acceptor-functionalized on PNCs. **S2**

2. Passivation effect of PNCs with PIC and TC. **S3**

3. Photoluminescence quenching of PNCs with QIC and BTC. **S4**

4. Functionalization of PNCs with a size of 5.5 nm with QIC **S5**

5. TA measurements of the 2ABS-purified and PIC/TC-passivated samples. **S6**

6. Time-Resolved Photoluminescence measurements of PNC samples **S7**

7. Evaluating the energy levels of QIC. **S6**

8. Phosphorescence measurements of QIC and PNCs with and without QIC. **S8**

References **S9**

1. Characterization of 2ABS-purified and acceptor-functionalized on PNCs.

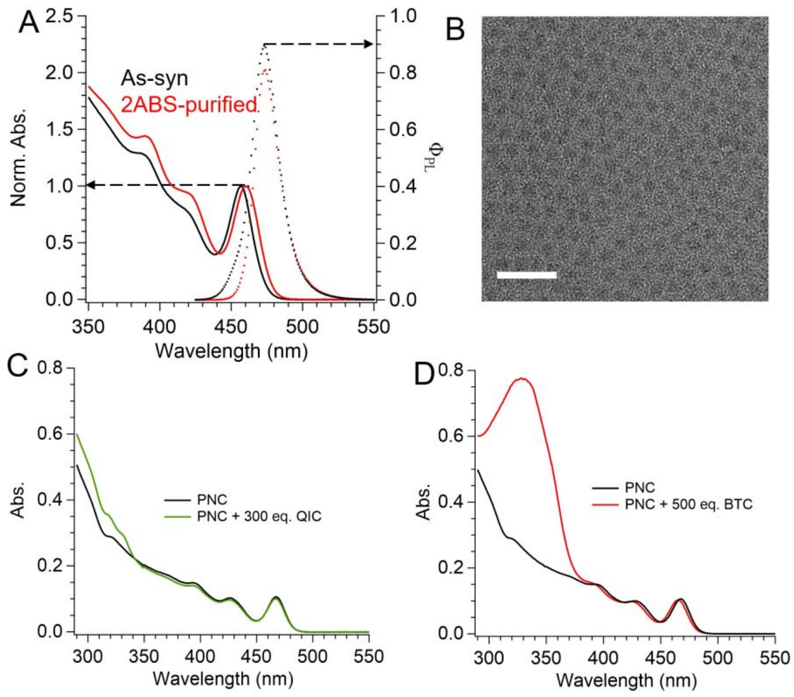

Figure S1. (A) UV-vis absorption and photoluminescence spectra PNC samples before (black) and after (red) the 2ABS purification. **(B)** TEM image of the PNC sample after 2ABS-purification. The scale bar represents 20 nm. **(C)** and **(D)** Comparison of UV-vis absorption spectra of 2ABS-purified samples with the addition of corresponding quenchers. The bandgap was not significantly affected by the purification process or by applying the quenchers, and thereby, the PNC integrity remained after all treatments.

2. Passivation effect of PNCs with PIC and TC.

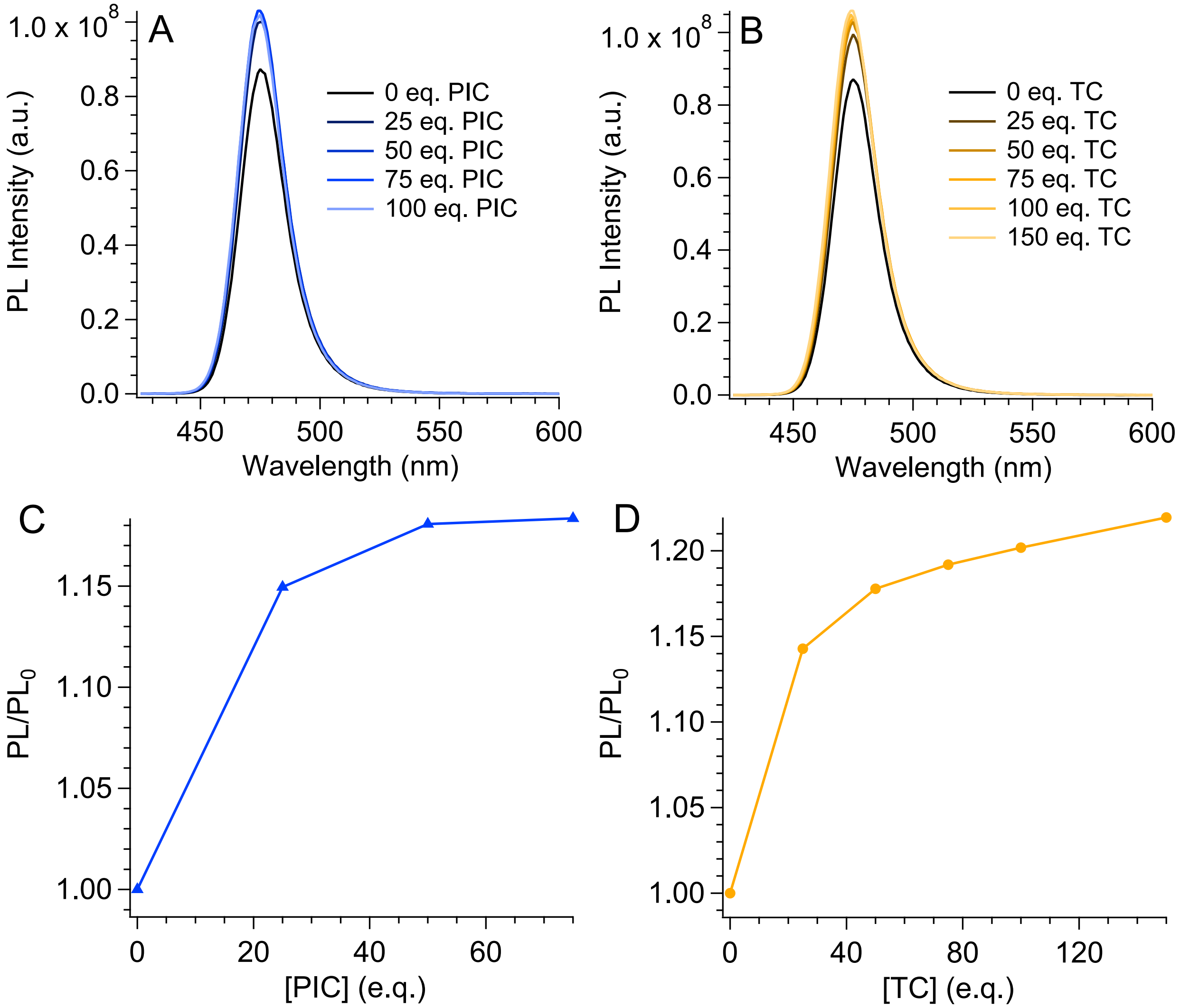

Figure S2. PL Spectra of the 2ABS treated PNCs in toluene with the addition of PIC **(A)** and TC **(B)** and the corresponding plots for the increasing PL intensities with **(C)** PIC and **(D)** TC passivation.

3. Photoluminescence quenching of PNCs with QIC and BTC.

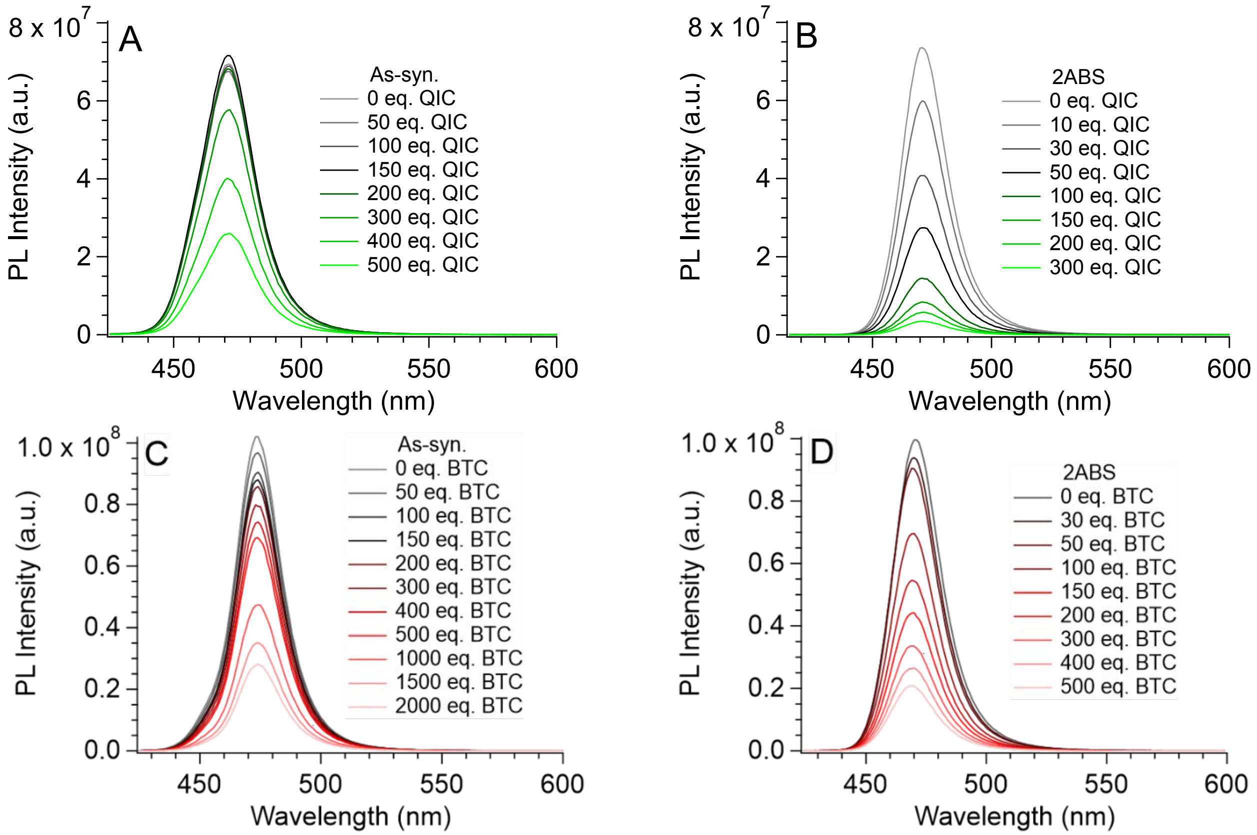

Figure S3. PL Spectra of the PNCs with the addition of QIC (green) and BTC (red) in toluene. The quenching of the as-synthesized PNCs (**A** and **C**), and 2ABS-treated PNCs (**B** and **D**) are displayed together to demonstrate the effectiveness of the surface reconstruction and its influence on both the binding equilibrium and, thereby, the electronic coupling.

4. Functionalization of PNCs with a size of 5.5 nm with QIC

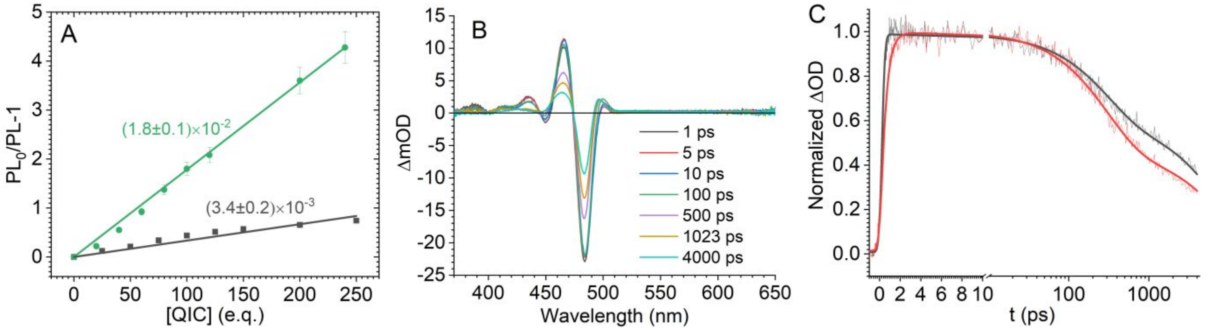

Figure S4. Applying the functionalization method to attach QIC to PNCs with a first excitonic absorption band at 484 nm. **(A)** PL quench plots of QIC when titrated to the as-synthesized (black), and 2ABS-purified PNCs (green). The fitting curves represent the linear fit of PL_0/PL-1 against the equivalent of the quencher. **(B)** TA spectra at selective time delays for 0.4 µM PNC in toluene with 400 e.q. QIC. The pump excitation wavelength was 420 nm. **(C)** Global fitting of the kinetic trace of the GSB at 484 nm and the PIA at 462 nm with shared decay rate constants as listed in **Table S1**.

Table S1. Global Fitting Results with Shared Time Constants (τ1 and τ2) from **Figure SX**.

Signal (nm)	τ_r (ps)*	τ_1 (ps), A_1	τ_2 (ns), A_2
GSB 484	--	300±20<br>0.40±0.01	7.8±0.6<br>0.60±0.01
PIA 462	0.49±0.02	300±20<br>0.54±0.01	7.8±0.6<br>0.46±0.01

*An exponential growth component is needed to fit the rise of the PIA-462 signal.

For the PNC with a first excitonic peak at 484 nm, our procedure demonstrated the same level of enhancement for the interaction between the PNC and the acceptor. QIC applied to the 2ABS-purified PNC showed a 6-fold increase in the quench rate constant. TA revealed a similar eT/CR mechanism between the PNC donor and the QIC acceptor. As illustrated by the kinetic fitting, with 400 e.q. of QIC, the eT rate constant was $(300\pm20\ ps)^{-1}$, and the CR rate constant was $(7.8\pm0.6\ ns)^{-1}$. It was worthwhile noting that the amplitude of eT and CR-related amplitudes in the GSB in this larger PNC were closer to the green-emission $CsPbBr_3$ (67.2% and 32.8%, respectively, as reported by ref. 45) compared to the small PNC shown in **Figure 4**. We attribute the evolution of the GSB kinetic to the diminished polaron effect in the larger PNCs.

5. TA measurements of the 2ABS-purified and PIC/TC-passivated samples.

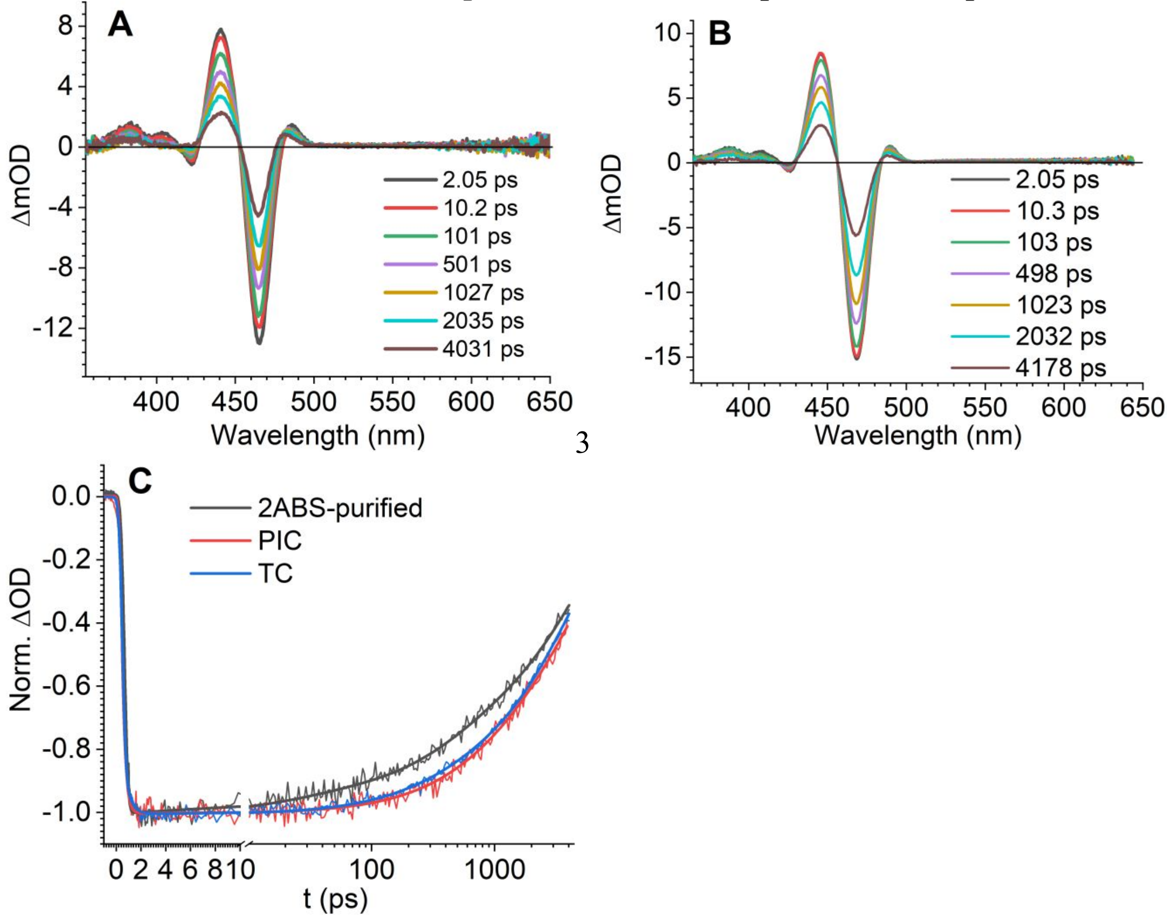

Figure S5. TA spectra at selective time delays for **(A)** the 2ABS-purified PNC and **(B)** Sample **(A)** passivated by 200 e.q. TC. **(C)** Comparison of kinetic traces of the GSB signals of the 2ABS-treated PNCs and the samples after PIC and TC-passivation. Notably, the initial fast decay kinetic component disappeared after passivating the sample with PIC and TC.

6. Time-Resolved Photoluminescence measurements of PNC samples

Time-resolved photoluminescence Details about the TRPL system can be found in ref.[1]. The pump excitation wavelength was 420 nm. 0.6 ns instrument response was determined according to the full-width-half-maximum of the pump scattering signal.

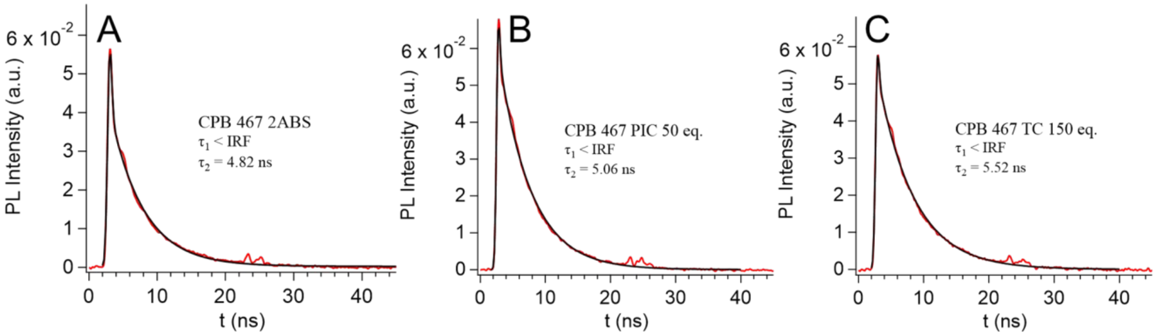

Figure S6. Time-resolved photoluminescence (TRPL) spectra of **(A)** 2ABS treated PNCs, **(B)** decorated with 50 eq. of PIC, and **(C)** 150 eq. of TC. Black curves are biexponential fitting for the experimental data. The fast component with a time constant <0.03 ns in the biexponential fitting is necessary to compensate for the instrument response jittering. The second components within the fits feature the actual photoluminescence lifetimes.

Evaluating the energy levels of QIC. The cyclic voltammetry experiment (**Figure S6.)** was performed using a CH Instruments electrochemical workstation along with a picoamp booster. The solvent was HPLC grade dichloromethane (DCM), dried with 4 Å molecular sieves overnight, with tetrabutylammonium hexafluorophosphate at 0.1 M concentration as the electrolyte. The electrochemical cell was composed of a glassy carbon working electrode, a Pt counter electrode, and an Ag/$AgNO_3$ (0.01M – acetonitrile) reference electrode. Due to the irreversible nature of QIC, we used the voltage obtained using the inflection potential, $E^{(i)}$, to determine the reduction potential.[2] The LUMO vs. vacuum was calculated as follows: $E_{LUMO} = V(Fc/Fc^+) – V(QIC^-) + (-4.76\ V)\ eV = 0.46\ V – (-1.07\ V) + (-4.76V) = -3.23\ eV$, where the -4.76 V is the potential of Fc/Fc^+ vs. vacuum.[3] The HOMO energy level was outside the voltage window of the DCM and was calculated using the optical bandgap (E_g =3.67 eV) of the molecule: $E_{HOMO} = E_{LUMO} – E_g = -3.23\ eV – 3.67\ eV = -6.9\ eV$.

7. Evaluating the energy levels of QIC.

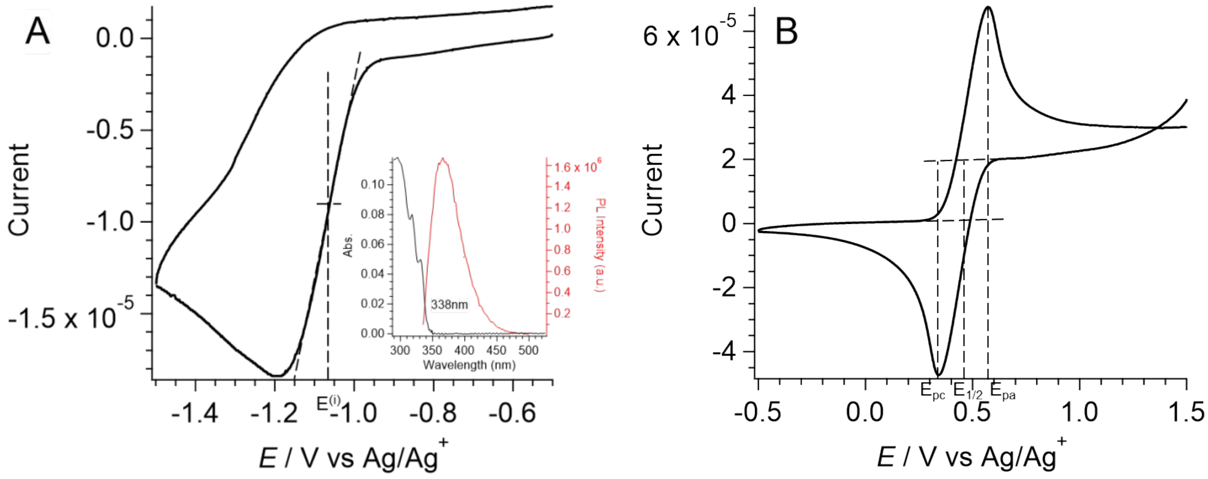

Figure S7. Cyclic voltammogram of QIC **(A)** with ferrocene **(B)** for the standard reference. Inset is the UV-vis absorption and the fluorescence spectra of QIC, which indicate E_{00} at 388 nm, and an optical bandgap of 3.67 eV.

8. Phosphorescence measurements of QIC and PNCs with and without QIC.

Phosphorescence Spectroscopy The phosphorescence spectra, as shown in **Figure S7,** were measured with a Horiba FluoroMax 3 fluorometer. The excitation wavelength for the QIC experiment was 330 nm, and for PNC and PNC + QIC spectra was 440 nm. The spectra were obtained at 77 K using a liquid nitrogen-filled dewar constructed with fused silica for the optical window. The samples were dispersed in toluene and measured using high-quality NMR tubes.

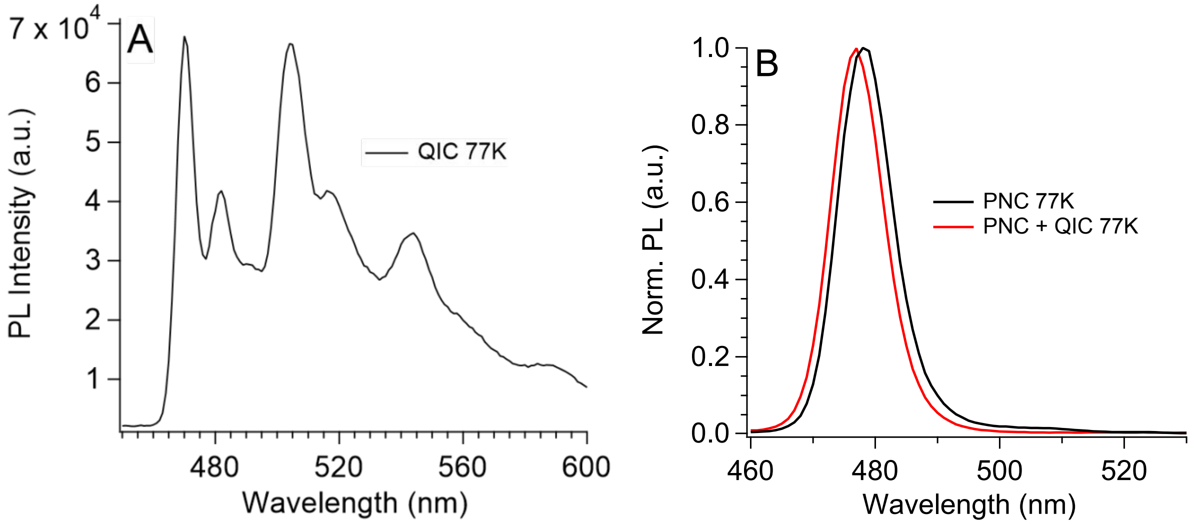

Figure S8. (A) Phosphorescence Spectrum of QIC, and **(B)** normalized PL spectra of the 2ABS purified PNC, and the PNC-QIC complex in toluene at 77 K.

References

1. Malinoski, A.; Hu, G.; Wang, C. Strong Bidentate Coordination for Surface Passivation and Ligand-Shell Engineering of Lead Halide Perovskite Nanocrystals in the Strongly Quantum-Confined Regime. *J. Phys. Chem. C* **2021,** 125, (44), 24521-24530. https://doi.org/10.1021/acs.jpcc.1c07952
2. Espinoza, E. M.; Clark, J. A.; Soliman, J.; Derr, J. B.; Morales, M.; Vullev, V. I. Practical Aspects of Cyclic Voltammetry: How to Estimate Reduction Potentials When Irreversibility Prevails. *Journal of The Electrochemical Society* **2019,** 166, (5), H3175. https://dx.doi.org/10.1149/2.0241905jes
3. D'Andrade, B. W.; Datta, S.; Forrest, S. R.; Djurovich, P.; Polikarpov, E.; Thompson, M. E. Relationship between the Ionization and Oxidation Potentials of Molecular Organic Semiconductors. *Organic Electronics* **2005,** 6, (1), 11-20. https://www.sciencedirect.com/science/article/pii/S1566119905000030
